# Neuromodulation of hippocampal long-term synaptic plasticity

**DOI:** 10.1016/j.conb.2018.08.009

**Published:** 2019-02

**Authors:** Jon Palacios-Filardo, Jack R Mellor

**Affiliations:** Centre for Synaptic Plasticity, School of Physiology, Pharmacology and Neuroscience, University of Bristol, Bristol BS8 1TD, UK

## Abstract

Multiple neuromodulators including acetylcholine, noradrenaline, dopamine and serotonin are released in response to uncertainty to focus attention on events where the predicted outcome does not match observed reality. In these situations, internal representations need to be updated, a process that requires long-term synaptic plasticity. Through a variety of common and divergent mechanisms, it is recently shown that all these neuromodulators facilitate the induction and/or expression of long-term synaptic plasticity within the hippocampus. Under physiological conditions, this may be critical for suprathreshold induction of plasticity endowing neuromodulators with a gating function and providing a mechanism by which neuromodulators enable the targeted updating of memory with relevant information to improve the accuracy of future predictions.

## Highlights

• Acetylcholine, noradrenaline, dopamine and serotonin all facilitate long-term synaptic plasticity.
• Neuromodulators facilitate long-term synaptic plasticity by common and divergent mechanisms.
• Common mechanisms include NMDA receptor facilitation by potassium channel inhibition, gliotransmission and disinhibition.
• Divergent mechanisms include diversity of disinhibition and temporal and spatial neuromodulator release.

**Current Opinion in Neurobiology** 2019, **54**:37–43

This review comes from a themed issue on **Neurobiology of learning and plasticity**

Edited by **Scott Waddell** and **Jesper Sjöström**

For a complete overview see the Issue and the Editorial

Available online 10th September 2018

**https://doi.org/10.1016/j.conb.2018.08.009**

0959-4388/© 2018 The Authors. Published by Elsevier Ltd. This is an open access article under the CC BY license (http://creativecommons.org/licenses/by/4.0/).

## Introduction

In competitive environments, it is important to make rapid accurate decisions in response to changing situations. This requires neuronal networks to accurately predict outcomes based on prior knowledge. The hippocampus plays a key role in the formation, updating and retrieval of memories which form the basis of outcome prediction. Memories are formed in the hippocampus by creating ensembles of strongly coupled neurons that encode a specific event or episode by the process of long-term synaptic plasticity which can be either potentiation (LTP) or depression (LTD). However, nowhere near all experienced events are turned into memories and therefore a filter operates to ensure only the most salient events are memorised. But what defines salience in this context? It is proposed that salience is signalled when a predicted outcome does not match the observed outcome leading to a state of uncertainty which may be reduced by updating the relevant memories [1,2]. Under this theoretical framework, uncertainty triggers the release of neuromodulators that act as the neurobiological filter controlling which memories are formed and updated [3]. Interestingly, the increase in uncertainty and release of neuromodulators often results from, and therefore occurs after, the salient event. This means that the mechanisms controlling synaptic plasticity need to incorporate an extended temporal window of interaction between a salient event and release of neuromodulator [4, 5, 6].

This review focuses on the mechanisms by which the neuromodulators acetylcholine, dopamine, noradrenaline and serotonin modulate long-term synaptic plasticity in the hippocampus, highlighting common and divergent themes. For more in-depth reviews on the action of each individual neuromodulator on synaptic plasticity please see [7, 8, 9, 10]. We propose that all the neuromodulators facilitate long-term synaptic plasticity often via common mechanisms but with differences potentially reflecting distinct aspects of uncertainty encoded by each neuromodulator.

## Acetylcholine

**Figure 1:**
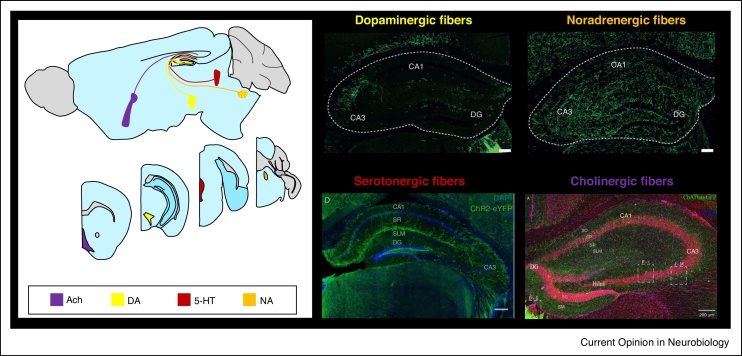
Neuromodulator projections to the hippocampus. (Left) Location of nuclei containing the cell bodies that give rise to neuromodulator projections for Acetylcholine (Ach), Dopamine (DA), Serotonin (5-HT) and Noradrenaline (NA). Sagittal (top) and Coronal (bottom) sections. Data from Allen Brain Atlas for mouse brain. (Right) Images of neuromodulatory projection fibers within the hippocampus labelled with eYFP targeted by viral injection into VTA and LC of TH-cre mice (top left and right respectively), YFP tagged channel rhodopsin in ePet1-cre:Ai32 mice (bottom left) and GFP in ChAT-tauGFP mice (bottom right). Images taken from Refs. [38^••^,65^••^,^77^]. Scale bars 200 μm.

Acetylcholine release in the hippocampus is prominently associated with learning and memory with acetylcholine depletion or cholinergic receptor blockade resulting in memory deficits. Cholinergic fibers from the medial septum/diagonal band of Broca release acetylcholine into the hippocampus (Figure 1) in response to arousal and primary reinforcement cues [11,12] which can also be described as expected and unexpected uncertainty [1].

**Figure 2:**
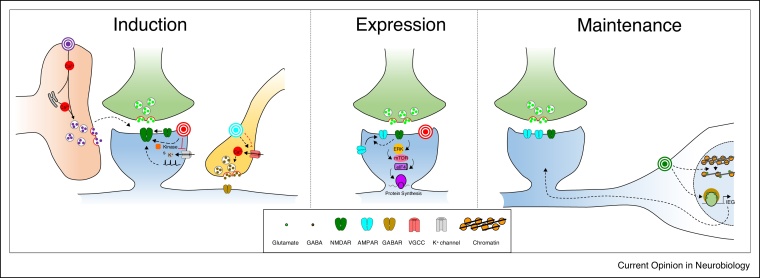
Mechanisms for neuromodulator facilitation of long-term synaptic plasticity. Major mechanisms for the regulation of long-term synaptic plasticity include: facilitation of induction by stimulation of astrocytes (purple), increased postsynaptic excitability and NMDAR activity (red) and disinhibition (blue); facilitation of expression by regulation of AMPAR function at the postsynaptic membrane (red); facilitation of maintenance by regulation of gene transcription (green).

Acetylcholine activates nicotinic ionotropic (principally α4β2, α3β4 and α7 in the hippocampus) and muscarinic (M1–4) G-protein coupled receptors (GPCRs) in different cell types and subcellular locations generating a myriad of potential signalling pathways. Increasingly, these pathways are seen to act in a coordinated manner to facilitate the induction and expression of LTP and LTD (Figure 2). Most of these pathways act to enhance NMDA receptor (NMDAR) activity but through several separate mechanisms. Firstly, muscarinic M1 receptors enhance postsynaptic excitability and NMDAR activity by inhibiting potassium channels including voltage activated Kv7 [13], Kv4.2 [14] and calcium activated SK channels [15,16] which facilitates the induction of LTP [13,16, 17, 18]. Secondly, acetylcholine also enhances NMDARs directly via a muscarinic receptor initiated signalling cascade where increased IP3 levels activate a series of protein kinases, including CAMKII, PKC and SRC, to facilitate induction of LTP [19] or through a direct interaction between nicotinic receptors and NMDARs [20]. However, it is reported that M1 receptors can also depress NMDAR activity through IP3 induced calcium release from internal stores resulting in dynamin dependent endocytosis of NMDAR [21]. Thirdly, a role for astrocytes has recently been uncovered where activation of astrocytic α7 nicotinic receptors causes the release of D-serine to enhance NMDAR activity and facilitate LTP induction [22^••^]. Fourthly, acetylcholine alters inhibitory interneuron activity and synaptic transmission with the potential to fundamentally reconfigure network excitability and favour plasticity at one set of excitatory synapses over another because subtypes of interneurons are able to precisely control specific neuronal dendritic regions. Due to the complexity of interneuron subtypes and functions and their responsiveness to acetylcholine, predicting the exact outcome of acetylcholine release is difficult but broadly an emerging view indicates that acetylcholine increases interneuron firing rates either directly [23] or via activation of astrocytes [24] and increases spontaneous GABA release [25]. However, evoked release of GABA is supressed by activation of presynaptic M2 receptors [26] or by postsynaptic activation of M1 receptors causing retrograde release of endocannabinoids [27]. Although the precise outcome of cholinergic modulation of inhibition is unclear a common feature is its transient nature, supporting a primary role in the induction of synaptic plasticity which is common to most of the mechanisms that facilitate NMDARs. There are a few exceptions to this rule where reports suggest that the expression of synaptic plasticity can also be facilitated by acetylcholine [19]. Furthermore, the facilitation of LTD by acetylcholine is likely via effects on the expression pathway and not through facilitation of NMDAR [21,28,29^••^].

Overall, the emerging picture indicates that acetylcholine facilitates LTP and LTD in the hippocampus and this facilitation becomes critical when LTP is induced by stimulation protocols that closely mirror endogenous activity patterns [17,28,29^••^,^30^] suggesting that in many situations acetylcholine is required for LTP induction *in vivo*.

## Noradrenaline

Noradrenaline release from fibers originating in the Locus Coeruleus (LC) (Figure 1) is strongly associated with novelty or salience [31], otherwise described as unexpected uncertainty [1], and, like acetylcholine, evidence indicates a role in the formation of memories since reduction in noradrenaline or blockade of noradrenergic receptors causes memory impairments [32].

Noradrenaline activates α1-adrenergic, α2-adrenergic and β-adrenergic GPCRs with the β receptors, signalling via Gs and cAMP, exerting the major impact on LTP and LTD. Noradrenaline facilitates LTP in CA1 [33, 34, 35] but there is also strong evidence for a role enhancing LTP and LTD within dentate gyrus — CA3 circuits [36,37] which links to evidence of increased density of noradrenergic fibers in these regions [38^••^]. There are several proposed mechanisms for the facilitation of LTP and LTD (Figure 2). Similar to acetylcholine, noradrenaline enhances NMDAR activity by inhibiting potassium channels including Kv1.1, Kv4.2 and SK channels [33,39,40] and by direct phosphorylation of NMDARs by PKA [41]. Compared to acetylcholine, there is a paucity of data on the actions of noradrenaline on inhibitory interneuron populations, so this area remains to be fully explored. Noradrenaline, via PKA, also phosphorylates AMPA receptors (AMPARs) promoting their insertion into the postsynaptic membrane [42,43]. PKA activation can also recruit protein synthesis [44] representing a delayed noradrenergic mechanism that can contribute to an expanded time window for modulation of LTP (Figure 2).

Despite strong *in vitro* and *in vivo* evidence supporting a role for noradrenaline in facilitating LTP and memory formation in the hippocampus, recent data suggests that dopamine rather than noradrenaline released by LC neurons is the important neuromodulator [38^••^,^45^,^46^]. This controversial finding fundamentally questions the role played by noradrenaline in hippocampal LTP and memory and will require further corroboration.

## Dopamine

Dopamine release has classically been associated with reward and prediction errors as well as novelty and salience that potentially lead to heightened uncertainty levels [2]. These ideas are principally based on dopamine release patterns in striatum from projections from the Ventral Tegmental Area (VTA) [47] and that selective stimulation of VTA dopaminergic projections induces synaptic plasticity and hippocampal-dependent behavioural changes [48,49^••^]. However, projections from the VTA are relatively sparse in the hippocampus with the suggestion that the major source of dopamine in the hippocampus actually arises from LC projections (Figure 1) [38^••^,^45^,^46^].

Dopamine exerts its action through 5 GPCRs clustered into two families: D1-like (D1 and D5) and D2-like (D2, D3 and D4) resulting in increased and decreased cAMP levels respectively. There is evidence for widespread expression of these receptors throughout the hippocampus but with some region-specific differences [50] and dimerization occurs between subtypes and with other neuromodulatory receptors including noradrenergic and cholinergic receptors making receptor pharmacology difficult to interpret [51]. Since the D1-like and D2-like receptors mechanistically act in opposition to each other, dopamine can both facilitate and depress synaptic plasticity but in general the D1 receptors appear to have most influence on plasticity processes decreasing overall cellular excitability but causing disinhibition [52] and modulation of NMDAR signalling [53] leading to facilitation of LTP and LTD (Figure 2) [54, 55, 56, 57, 58]. However, stimulation of endogenous dopamine release from VTA projections can produce different effects to exogenous dopamine agonists [49^••^] questioning the physiological relevance of pharmacological receptor activation.

Dopamine can also facilitate LTP and LTD when the receptors are activated after a plasticity inducing event (often with a considerable delay) [54] via engagement of gene transcription and protein translation (Figure 2). The proposed mechanism suggests that translated proteins are then translocated to synapses primed for plasticity by coincident stimulation [59]. Recent evidence also suggests a role for dopamine in the long-term regulation of inhibition in CA3 which is important for memory consolidation [60] and combines a delayed action for dopamine with changes to the inhibitory network. Such mechanisms are attractive because they align the timescales of behavioural learning with the cellular mechanisms of synaptic plasticity where reward, novelty and salience are often determined after the event.

## Serotonin

Serotonin has classically been associated with mood but recent evidence suggests a prominent role in adaptation and plasticity where serotonin release occurs in response to prediction errors thereby promoting learning in conditions of uncertainty [61]. Serotonin is released by fibers projecting diffusely from the median raphe nucleus (MRN) into all parts of the hippocampus (Figure 1).

Serotonin (5-HT) receptors are composed of seven families (5-HT_1_ to 5-HT_7_) which are coupled to GPCRs except for the ionotropic 5-HT_3_ receptors and all 5-HT receptors are expressed in the hippocampus. Serotonin has been shown to increase cellular excitability by several mechanisms (Figure 2), such as inhibition of K^+^ channels [62], and serotonin also enhances NMDAR activation [63] and facilitates LTP expression in CA1 [64,65^••^,^66^]. Stimulation of interneurons and astrocytes by activation of 5-HT receptors has been reported [67,68] but it is not yet clear whether this can also facilitate LTP. Conversely, 5-HT is mainly inhibitory in CA3 and prevents LTD or LTP at mossy fiber-CA3 synapses [69] highlighting the cell specific nature of serotonergic signalling.

## Similarity and divergence across neuromodulatory systems

The neuromodulators acetylcholine, dopamine, noradrenaline and serotonin have historically been viewed as distinct systems signalling different behavioural states but increasingly this compartmentalised perspective is being broken down as overlaps in function and behavioural response are found. Observations of neuromodulator co-release [38^••^] and co-activation of receptor populations fundamentally questions this discrete model for neuromodulator function. Moreover, an alternative overarching theoretical framework suggests that each neuromodulatory system encodes aspects of uncertainty signalling the need to update memory representations to make better future predictions and reduce uncertainty [1,2]. A central prediction of this theoretical framework is that the release of these neuromodulators facilitates long-term synaptic plasticity in the hippocampus. In this review we outline evidence to support this prediction where each neuromodulator triggers mechanisms that facilitate LTP and/or LTD. Thus, there is considerable support for a common function for the separate neuromodulatory systems and indeed for some common mechanisms (e.g. enhancing NMDAR activity) but there are also evident differences.

Three potential mechanistic similarities for NMDAR enhancement are highlighted (Figure 2). The first is the pivotal role played by SK channels which control spine NMDAR activity and can be inhibited by acetylcholine, noradrenaline and metabotropic glutamate receptors [15,16,40,70] and may also be inhibited by other neuromodulators although this has yet to be tested. The inhibition of SK channels leads to the gating of LTP induction. The second is reconfiguration of interneuron activity and inhibitory transmission thereby differentially altering excitability and activation of NMDARs along dendrites and across cell types. The complexity of the interneuron network makes unravelling the impact of neuromodulators difficult, but data is emerging for several of the neuromodulators considered here [23,52,67] and it will be interesting to see how the reconfiguration of hippocampal interneuron networks modulates LTP and LTD. The third is the role played by astrocytes potentially coupling circadian rhythms of neuromodulator release to induction of synaptic plasticity. This has been demonstrated for acetylcholine [22^••^] and there is good evidence for excitation of astrocytes by noradrenergic, dopaminergic and 5-HT receptors [68,71,72]. Another common feature of the neuromodulators is the facilitation of both LTP and LTD (although often at different synapses) which may reflect the importance of both forms of plasticity for memory formation and consolidation.

Three divergent mechanisms are also highlighted (Figure 2). Firstly, the common mechanism of reconfiguring interneuron networks and inhibitory transmission may vary considerably in the details. For example, acetylcholine excites somatostatin and parvalbumin expressing oriens-lacunosum-moleculare interneurons decreasing distal dendritic excitability in CA1 pyramidal neurons [23] whereas serotonin excites cholecystokinin expressing interneurons at the stratum radiatum/lacunosum moleculare border causing widespread inhibition [67]. Secondly, although not described in this review, the precise hippocampal regions and synapses for each neuromodulatory mechanism vary considerably. Interestingly, this may mirror the differential innervation of hippocampal regions by neuromodulator projections both in terms of subregions (e.g. dentate gyrus, CA3, CA1) and the longitudinal axis of the hippocampus dorsal to ventral where these regions perform distinct tasks and are differentially innervated by neuromodulators (Figure 1). As an example, the expression of SK channels in CA1 pyramidal cells (and therefore the regulation of NMDARs and threshold for LTP induction) is higher in the ventral than dorsal hippocampus [73^••^] providing a mechanism for differential control of LTP by neuromodulators along the dorsal–ventral axis. Finally, as well as spatial differentiation, there is evidence that the different neuromodulator systems may vary in their ability to facilitate plasticity on a delayed timescale. Dopamine [54,59,60] and to a lesser extent noradrenaline [42, 43, 44] can act on delayed timescales providing a retroactive mechanism for facilitating synaptic plasticity which has also been demonstrated at synapses in the neocortex and striatum [74, 75, 76]. Conversely, acetylcholine and serotonin act principally on shorter timescales to facilitate induction of plasticity. This implies that acetylcholine and serotonin play a greater role signalling expected uncertainty and create a prolonged state of arousal during which plasticity is facilitated. Conversely, dopamine and noradrenaline signal unexpected uncertainty and create a transient arousal that signals retroactively to facilitate plasticity in response to recent events [1,2]. These considerations raise the intriguing question of how separate neuromodulator systems interact to control plasticity [29^••^].

## References and recommended reading

Papers of particular interest, published within the period of review, have been highlighted as

•• of outstanding interest
